# Exploring the Impact of a Digital Reading Program on Apathy Among Community-Dwelling Older Adults in Rural Canada: Insights from Socioemotional Selectivity Theory

**DOI:** 10.3390/geriatrics11010001

**Published:** 2025-12-24

**Authors:** Aderonke Agboji, Shannon Freeman, Davina Banner, Joshua Armstrong, Melinda Martin-Khan, Alexandria Freeman-Idemilih

**Affiliations:** 1 School of Nursing, University of Northern British Columbia, Prince George, BC V2N 4Z9, Canada; shannon.freeman@unbc.ca (S.F.); davina.banner-lukaris@unbc.ca (D.B.); 2 Department of Health Sciences, Lakehead University, Thunder Bay, ON P7B 5E1, Canada; jjarmstr@lakeheadu.ca; 3 Health and Community Sciences, University of Exeter, Exeter EX1 2LU, UK; m.martin-khan@exeter.ac.uk; 4 Faculty of Health, University of Waterloo, Waterloo, ON N2L 3G1, Canada; ancvfreemanidemilih@uwaterloo.ca

**Keywords:** older adult, apathy, eBook club, eReaders, socioemotional selectivity theory, mixed-methods study

## Abstract

**Background/Objectives:** Apathy, characterized by diminished motivation and reduced engagement in goal-directed behavior, is a prevalent concern among older adults, particularly in rural communities where opportunities for meaningful engagement may be limited. This study explores the preliminary impact of an in-person eBook club program on apathy among community-dwelling older adults in Northern British Columbia. **Methods:** This eight-week pilot single-group, pre-post mixed-methods study combined the use of eReaders to access weekly reading materials with facilitated in-person group discussions designed to foster emotional and social connection. Apathy was assessed using the 3-item Geriatric Depression Scale (GDS-3A) before and after the program. **Results:** A Wilcoxon signed-rank test revealed a statistically significant reduction in apathy scores (Z = −4.01, *p* < 0.001), with a large effect size (r = 0.76). While not powered for hypothesis testing, these findings suggest the program may have a meaningful effect. Qualitative analysis of participants who reported higher baseline apathy scores identified three key mechanisms of change: positivity effect, selective pruning of social networks, and adaptive coping, consistent with socioemotional selectivity theory. **Conclusions:** These preliminary results support the feasibility and potential value of theory-informed, low-cost group reading programs for addressing apathy in older adults and can inform the design of a larger, controlled study.

## 1. Introduction

Aging is a global demographic trend that presents both challenges and opportunities for health and social systems [1]. In rural Canada, the growth in the older adult population continues to grow steadily. For example, in the Cariboo region of Northern British Columbia (BC), individuals aged 65 years and older accounted for 16.5% of the population in 2021, with significant growth expected through 2046 [2]. These trends underscore the increasing need for innovative strategies to support healthy aging in rural and underserved communities.

Apathy, characterized by diminished motivation and engagement in goal-directed behavior, is a significant concern for community-dwelling older adults [3,4,5,6,7]. It is often underrecognized or misattributed to normal aging, dementia, or depression, despite evidence that apathy is a distinct clinical syndrome requiring targeted intervention [4,8,9] with research consistently linking it to an elevated risk of cognitive decline, physical frailty, and adverse psychosocial outcomes. For example, one study found that severe apathy in older adults was associated with a 70% increased risk of developing dementia [10], while another study identified it as a predictor of both motoric cognitive risk syndrome and mild cognitive impairment [11]. Some studies have reported associations between apathy and reduced physical activity [12], slower gait speed, and diminished capacity to perform activities of daily living [13,14]. From a social perspective, apathy has been shown to contribute to increased isolation, decreased community engagement, and a greater burden on caregivers [15,16], highlighting the detrimental effects of apathy on older adults’ health, functioning, and social well-being.

While pharmacological treatments for apathy remain limited, non-pharmacological interventions that offer social, emotional, and cognitive engagement have shown promise [17,18]. Programs such as discussion groups, arts activities, and reminiscence therapy have been shown to foster purpose and connection among older adults with and without dementia in the community and long-term care settings [17,18,19]. Although studies examining reading as a direct intervention for apathy are sparse, extant literature suggests that reading enhances emotional well-being, cognitive stimulation, and reduced social withdrawal (factors closely associated with apathy in older adults) [9,19,20,21,22]. Additionally, digital reading platforms (e.g., eReaders, tablets, or iPads) have been linked to improved access to cognitive and social engagement opportunities among individuals with sensory or mobility challenges and those in remote or rural settings [23,24,25,26]. Shared reading initiatives, including book clubs, represent a particularly low-cost and scalable approach to improving engagement and social interactions among older adults, particularly those with dementia [27,28]. However, the structured use of eBook clubs as an intervention for apathy remains underexplored, especially in rural communities with limited social infrastructure.

Socioemotional selectivity theory (SST) provides a valuable framework for designing interventions that foster social, emotional, and cognitive engagement in later life. According to SST, as individuals perceive their remaining time as more limited with age, they shift their priorities toward emotionally meaningful goals and relationships [29,30]. This motivational shift influences how older adults allocate their attention, memory, and social effort, giving rise to the “positivity effect” ([31], p. 496), a tendency to focus more on emotionally positive than negative stimuli. In line with this shift, older adults often engage in selective narrowing of their social networks to prioritize close, rewarding connections [32]. They may also rely on emotionally meaningful activities as a form of adaptive coping in response to age-related physical or cognitive decline [33]. Activity programs grounded in SST can therefore support sustained motivation and psychological well-being by aligning with the intrinsic goals and values of older adults [34].

Despite SST’s theoretical relevance, few studies have explicitly applied its principles, and even fewer have targeted older adults in rural contexts. Thus, the aim of this study is to examine the impact of a structured digital reading program (henceforth referred to as eBook club) on apathy among community-dwelling older adults and to explore the underlying mechanisms of observed change.

## 2. Materials and Methods

### 2.1. Study Design

This pilot study employed a single-group, pre-post, convergent mixed-methods design [35] to evaluate the preliminary impact of an eBook club program on apathy in older adults residing in rural Northern British Columbia (BC), Canada. Quantitative and qualitative data were collected concurrently and analyzed in an integrated manner to develop a comprehensive understanding of participants’ experiences and outcomes. In line with established guidelines for pilot studies, no formal sample size calculation was conducted, as the primary aim was to gather preliminary data on the program’s effects rather than to test hypotheses or estimate treatment efficacy [36,37]. However, we aimed to recruit approximately 30 participants based on feasibility considerations. This number was deemed appropriate to allow for preliminary examination of outcomes and variability while informing the design of future trials. For the qualitative component, we targeted 12–15 semi-structured interviews, guided by the principle of data saturation [38]. While the single-group design limits internal validity, it was deemed appropriate for this early stage of program development [36].

Demographic information was collected using a semi-structured interview guide (see Supplementary Materials) administered before the intervention by the first author. Participants self-reported their age, gender identity, marital status, highest level of education, and whether they had received a formal dementia diagnosis. Education was initially collected using four categories (less than high school, high school completion, college/university degree, and postgraduate studies). For analysis, these responses were consolidated into three categories to improve interpretability: high school, college/university, and other. All demographic variables, including age, gender, marital status, education, and dementia diagnosis, were self-reported.

### 2.2. Participants and Recruitment

This study was conducted in three rural communities in Northern British Columbia (BC), with the program lasting eight weeks in each community (March to September 2023). Participants were recruited using purposive and snowball sampling through social media (Facebook), community bulletin boards, and word of mouth. Participants were eligible if they (1) were aged 60 years or older and lived in rural communities in Northern BC; (2) were fluent in English; (2) had the cognitive ability to engage in reading activities; and (3) had access to an eReader device. A formal clinical diagnosis of apathy was not required to allow for the inclusion of individuals with varying levels of motivational decline, including subclinical apathy, which is particularly relevant for early, community-based interventions. Individuals with a formal diagnosis of severe cognitive impairment or late-stage dementia were excluded. Informed consent was obtained from all participants prior to enrollment.

Ethical approval for the study was granted by the Harmonized Ethics Review process in collaboration with the Institutional Research Ethics Board. Given the inclusion of participants with mild to moderate cognitive impairment, including those with a formal diagnosis of dementia, capacity to consent was assessed using the University of California, San Diego Brief Assessment of Capacity to Consent (UBACC) [39]. The UBACC was administered individually by the first author to evaluate participants’ understanding of the study’s purpose, procedures, potential risks, and benefits, and their ability to voluntarily provide informed consent. For participants with dementia, additional care was taken to ensure responses reflected meaningful comprehension, and where necessary, input was sought from caregivers or substitute decision-makers in accordance with ethical guidelines. The UBACC assessment was repeated prior to each data collection point to monitor ongoing capacity and reaffirm voluntary participation. All participants were informed of their right to withdraw at any time without consequence. Confidentiality was maintained through the assignment of anonymized identification codes to all data collected.

### 2.3. Apathy Measures

As aforementioned, apathy was measured using a self-report questionnaire known as the Apathy Subscale of the Global Depression Scale (GDS or GDS-3A) derived from the GDS-15. The GDS-3A consists of three questions, with scores ranging from 0 to 3, where higher scores indicate greater apathy. The scale was administered before the implementation of the eBook club and at eight weeks post-implementation of the eBook club. These three items included: “Have you dropped many of your activities and interests?”; “Do you prefer to stay in your room rather than going out and doing new things?”; and “Do you feel full of energy?” The scoring method for these questions was as follows: for questions 1 and 2, a “Yes” response was given a score of 1, while for question 3, a “No” response was given a score of 1. The GDS-3A has been widely used in research [34,40,41,42,43]. The validity of the GDS-3A has been evaluated for its sensitivity, ranging between 29.3% and 69%, and specificity, ranging between 85% to 92.6% [40], suggesting that the GDS-3A is a valuable tool for screening apathy in older adults [40]. The GDS-3A was selected for this study because it is a brief, easily administered tool suitable for older adults in community settings, some of whom might find longer assessments burdensome [44]. A cut-off score of ≥2 on the GDS-3A was used to indicate the presence of apathy, based on prior research linking this threshold to clinically significant apathy symptoms in older adults [40]. Accordingly, scores of 0 or 1 were categorized as ‘non-apathetic’, while scores of ≥ 2 were classified as ‘apathetic’.

### 2.4. Description of the eBook Club Program

**Figure 1:**
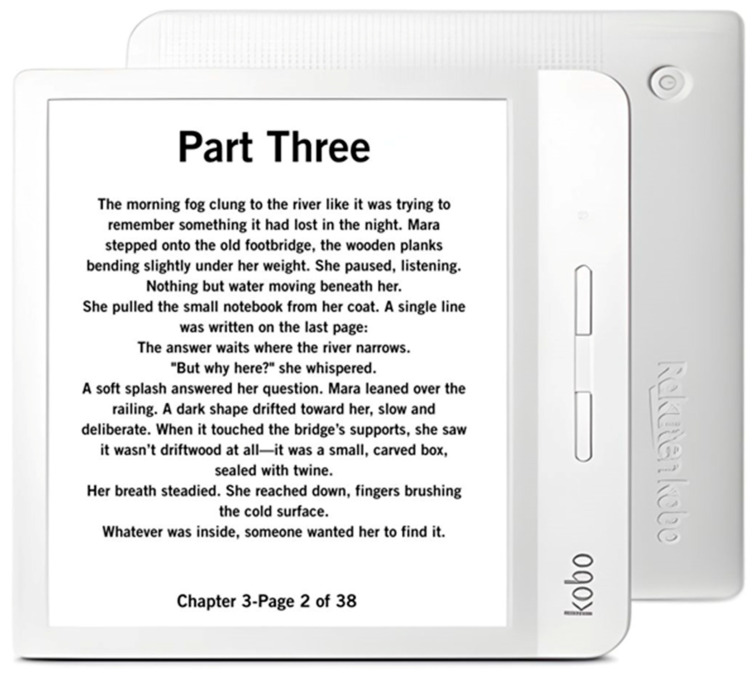
Kobo Libra H20 eReader device used during the program, displaying a typical fiction text. Participants engaged in both independent and group-based reading using this device throughout the 8-week program.

The eBook club consisted of weekly in-person group reading sessions supplemented by independent reading. Sessions were held at public libraries in each study site, progressing sequentially across the three communities. The program was delivered over eight weeks per site, with this duration determined by seasonal considerations and participant availability. To accommodate participants’ summer travel schedules and maximize engagement, the program timeline was adjusted accordingly. For group reading sessions, books were selected collaboratively during the pre-intervention phase based on participants’ interests and reading preferences, as assessed in initial interviews. Materials used in the programs included Kobo eReaders (Kobo Libra H20, Rakuten Kobo Inc, Ontario, Canada) (see Figure 1), the Rakuten Kobo eReader platform: www.kobo.com (accessed 5 March 2023) for creation of free Kobo accounts, access to the public library via Overdrive app, pre-loaded or downloadable eBooks, reading trackers for independent readers, as well as participant information letters, consent forms, and pre/post-interview protocols. All participants were provided with Kobo eReaders at no cost during enrollment. This helped ensure that lack of access to a device was not a barrier to participation. For those who preferred using their own devices, such as smartphones, tablets, or iPads, the Kobo app was supported and available, offering flexibility based on participants’ comfort and familiarity with technology. Participants were also responsible for their own travel to the in-person sessions.

**Figure 2:**
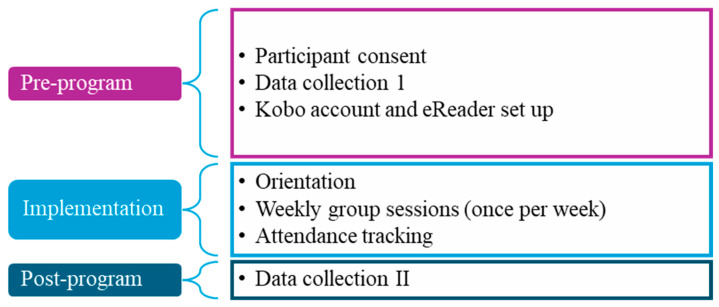
This figure illustrates the structure of the digital reading program, outlining the sequence of activities across pre-program, implementation, and post-program phases.

The procedures followed a three-phase structure: pre-program, implementation, and post-program (Figure 2). During the pre-program phase, participants completed a semi-structured interview that assessed reading preferences and choice of books to read. Kobo accounts were created, and eBooks were either pre-loaded on Kobo devices or made available via the Kobo app on personal devices. Technical support was provided as needed. Weekly group reading sessions, facilitated by the first author and a trained research assistant (sixth author), were held once a week for eight weeks in each community, with each session lasting 45 to 60 min. Importantly, the program was designed to build on existing social relationships. As a result, family members and friends were invited and encouraged to participate, supporting their loved ones during reading sessions and engaging in discussion. In addition, participants were given the opportunity to form new but emotionally resonant connections within a supportive context. This approach aligns with SST, which posits that older adults prioritize emotionally meaningful relationships as they age. Attendance ranged from four to eight participants per session, providing a supportive environment for engaged discussion and shared reading experiences.

**Figure 3:**
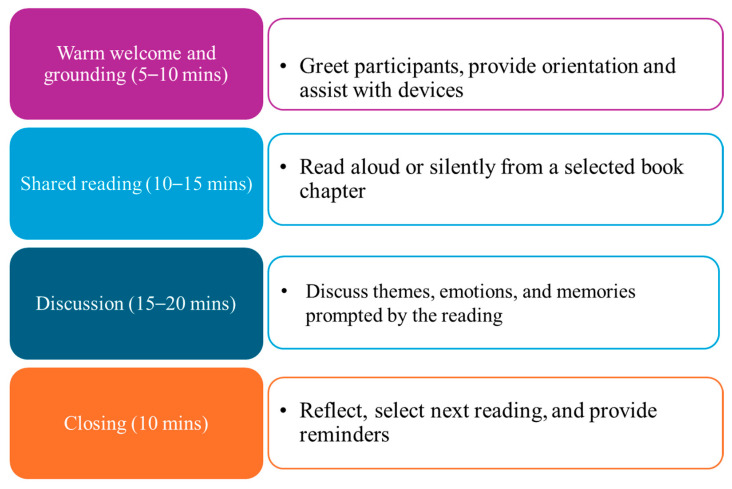
This figure shows activities during weekly sessions.

Fidelity was primarily assessed through systematic attendance tracking. Trained research assistants recorded participant attendance at each weekly session using standardized attendance logs. Figure 3 shows activities during weekly sessions. Consistent participation across sessions was considered a key indicator of intervention exposure. In addition, informal observations were conducted by facilitators to ensure consistency in session delivery across groups, including the structure of discussions, participant engagement, and adherence to the reading schedule. While a formal fidelity checklist was not used, facilitators followed a standardized session guide to maintain consistency in the implementation.

### 2.5. Data Collection

Data collection occurred at two time points: baseline (week 1) and immediately following the program (week 8). Quantitative data were collected using GDS-3A, while qualitative data were gathered via two semi-structured interviews by the first author. The first semi-structured interviews, conducted after informed consent was obtained, collected demographic information, reading preferences, and initial thoughts on using eReaders, establishing a baseline understanding of participants’ backgrounds and expectations. The second semi-structured interviews were conducted post-program to explore participants’ experiences with the eBook club. Accordingly, interview questions emphasized participants’ reasons for taking part, how the activity aligned with their goals and values, and whether the eBook club supported continued engagement in reading as a meaningful pursuit. The open-ended, semi-structured format allowed participants to freely express their experiences while ensuring that SST-relevant themes such as motivation, prioritization of meaningful activity, and perceived benefits were consistently addressed.

### 2.6. Data Analysis

Quantitative and qualitative data were analyzed independently and integrated during interpretation, following a convergent mixed-methods approach [35]. Descriptive statistics for participant demographics, including age, sex, education level, marital status, and dementia diagnosis, were compiled using Microsoft Excel to summarize sample characteristics. All quantitative analyses were conducted using the Statistical Package for the Social Sciences (SPSS, Version 29) [45], with statistical significance defined as *p* < 0.05. Given the ordinal nature of the GDS-3A and the small sample size, medians and interquartile ranges (IQRs) were calculated. To assess within-subject changes in apathy scores from pre- to post-program, the Wilcoxon signed-rank test was employed. This non-parametric test is suitable for paired ordinal data and does not assume normality, making it appropriate for evaluating median differences in small samples [46,47]. Subgroup analyses were conducted to explore differences in program response between participants with and without a dementia diagnosis. Due to the limited number of participants in the dementia subgroup, these analyses were descriptive and intended to generate hypotheses rather than support inferential conclusions. The attendance rate was also analyzed. The attendance rate was calculated as the number of sessions attended divided by the total number of sessions offered (8 sessions), expressed as a percentage.

All qualitative interviews were audio-recorded, transcribed verbatim, and anonymized to ensure participant confidentiality. Data were analyzed using thematic analysis, following the six-phase process outlined by Braun and Clarke (2006) [48]: familiarization with the data, generating initial codes, searching for themes, reviewing themes, defining and naming themes, and producing the report. Thematic analysis is widely recognized as a flexible and rigorous approach, well-suited for use within mixed-methods research designs [49]. NVivo 12 software [50] was used to organize, code, and support the analytic process. While the coding approach was primarily inductive, allowing themes to emerge from the data, final interpretation was informed by SST to position findings within a motivational framework relevant to later life. Following independent analysis, qualitative and quantitative results were integrated using triangulation to offer a comprehensive understanding of the program’s impact.

### 2.7. Methods to Ensure Trustworthiness and Rigor

Multiple strategies were employed to enhance the trustworthiness and rigor of both the quantitative and qualitative components of the study. In the quantitative component, steps were taken to ensure data accuracy, consistency, and appropriate analytic application. Firstly, all quantitative data were independently verified by two researchers (first and second authors) to confirm accuracy prior to analysis. This manual cross-checking process reduced the risk of data entry errors and ensured that subsequent statistical results were based on validated information [51]. Secondly, appropriate non-parametric statistical methods were selected based on the scale and distribution of the data. Apathy was measured using the GDS-3A, which yields ordinal scores. To assess pre- and post-program changes in self-reported apathy, the Wilcoxon signed-rank test was employed, as it is suitable for analyzing paired ordinal data in small samples without assuming normality [51]. Thirdly, descriptive statistics including medians, interquartile ranges (IQRs), means, and standard deviations were calculated to summarize the central tendencies and variability of the data. These measures provided a fuller understanding of the data distribution and supported interpretation beyond reliance on statistical significance alone [52].

In the qualitative component, investigator triangulation was used to enhance the reliability and credibility of the findings. Two independent researchers (first and second authors) conducted the initial coding and thematic analysis of interview transcripts. Discrepancies in coding were discussed and resolved through peer debriefing, where all the authors critically examined the emerging themes to ensure that they accurately represented participant experiences [53]. In addition, thick description and reflexivity were applied to enhance the depth and transparency of the qualitative findings. Thick description involved detailed accounts of participant experiences, incorporating direct quotes to provide contextual richness and illustrate key themes. Reflexivity was maintained by documenting researcher assumptions, biases, and analytic decisions in an audit trail, ensuring that interpretations remained grounded in participant narratives and not influenced by preconceived expectations [54].

## 3. Results

### 3.1. Sample Characteristics

**Figure 4:**
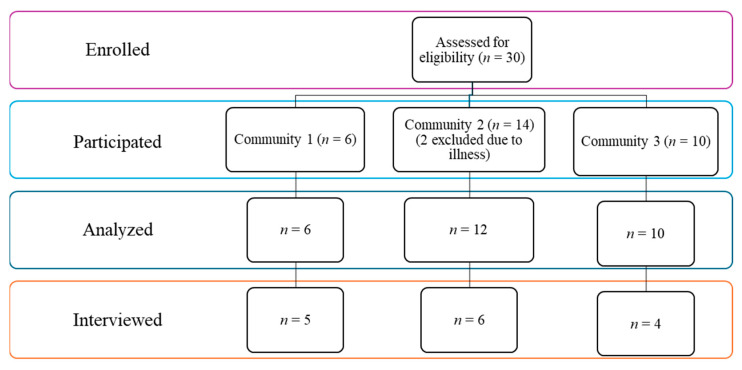
Participant flow diagram across the three rural communities. Of the 30 individuals assessed and enrolled, all participated in the intervention (Community 1: *n* = 6; Community 2: *n* = 14; Community 3: *n* = 10). Two participants from Community 2 were excluded from the final analysis due to illness, resulting in a total of 28 participants analyzed. 15 participants completed post-intervention interviews.

Table 1 presents the sample characteristics. A total of 28 community-dwelling older adults were included in the final analysis, following the withdrawal of two participants due to illness (Figure 4). The mean age of participants was 72.82 years (SD = 6.54), with an age range of 60 to 86 years. The majority (86%) were between 60 and 80 years old. Most participants identified as women (89%). In terms of educational attainment, 50% had completed high school, while 46% held a college or university degree. A smaller proportion (4%) had completed education below high school level. Most participants were married (57%), while others were divorced, widowed, or never married. Three participants (11%) had a formal diagnosis of dementia. With respect to reading habits, 71% of participants self-identified as regular readers. Fiction was the most preferred genre, with 75% reporting an interest in various forms of fictional literature, including travel adventure, romance, mystery, and blended genres. Approximately one-third of the sample regularly used digital devices such as smartphones, tablets, or eReaders for reading. Notably, 32% of participants reported no prior experience with digital reading technologies before the program.

### 3.2. Impact of the eBook Club on Apathy

Descriptive statistics for pre- and post-program apathy scores, measured using the GDS-3A, are presented in Table 2. The mean pre-program apathy score was 1.54 (SD = 1.20), which decreased to 0.64 (SD = 0.75) post-program. Median scores also declined from 2.00 to 1.00, with a reduction in interquartile range from 2.00 to 1.00.

**Figure 5:**
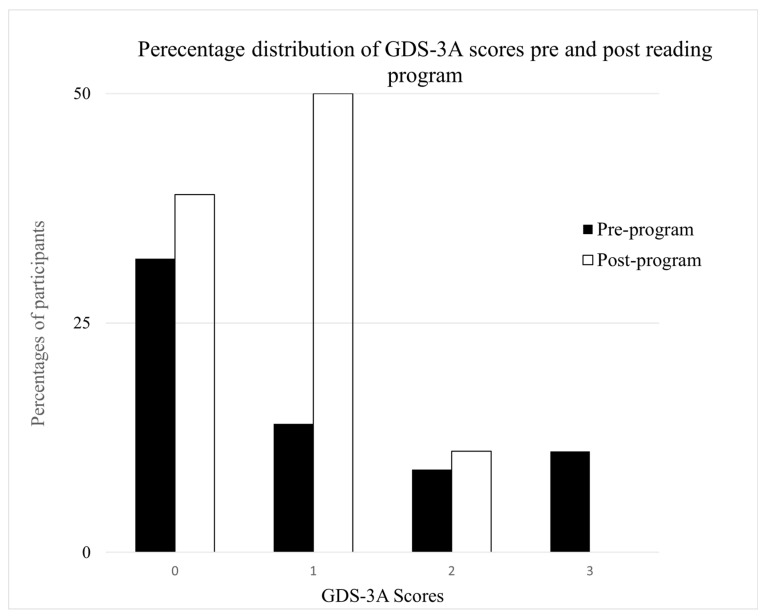
This figure shows a shift in GDS-3A scores before and after the eBook club program. The percentage of non-apathetic participants (score = 0 or 1) increased notably, while those scoring 2 or 3 declined from 50% to 7.1%, indicating a clear reduction in self-reported apathy.

**Figure 6:**
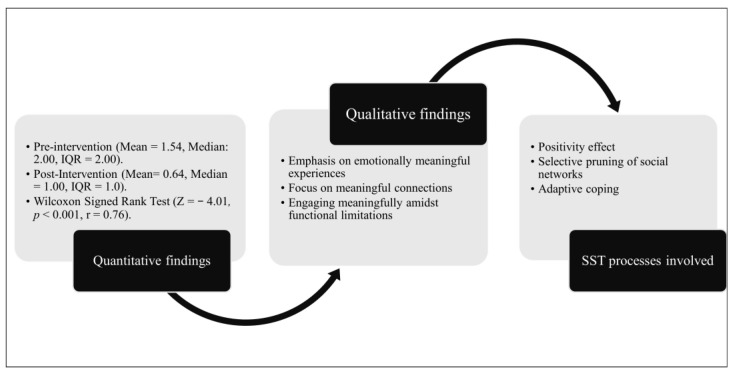
Joint display of quantitative and qualitative findings illustrating how quantitative reductions in apathy scores align with qualitative themes and reflect the core processes of SST involved.

Figure 5 illustrates the distribution of GDS-3A scores before and after the program, and Figure 6 presents a joint display integrating quantitative and qualitative findings. The results reveal a marked shift toward lower apathy scores following the program. Specifically, the proportion of participants with a score of 0 (indicating non-apathetic) increased from 36% at baseline to 43% post-program. The most substantial change occurred among those who scored 1, rising from 14% pre-program to 50% post-program. Additionally, the percentage of participants with scores of ≥2 decreased post-program (from 50% pre-program to 7% post-program). A Wilcoxon signed-rank test was also conducted to assess the change in apathy scores across the group (Figure 5). Results indicated a statistically significant reduction in apathy from pre- to post-program (*Z* = −4.01, *p* < 0.001). The effect size was large (*r* = 0.76), suggesting a substantial and clinically meaningful improvement in apathy following participation in the program. The average attendance rate per community was 98%.

To understand the mechanisms underlying these changes, qualitative data from participants who self-reported apathy pre-program (*n* = 15; apathy scores of ≥2) were analyzed. Three central themes emerged, each mapped onto well-established principles of SST: (1) positivity effect, (2) selective pruning of social networks, and (3) adaptive coping. Each theme is described as follows:

#### 3.2.1. Theme 1: Positivity Effect

The first primary theme reflected the SST principle of the positivity effect, wherein older adults selectively focus on emotionally gratifying experiences to optimize emotional well-being, resulting in decreased apathy scores. In accordance with this prediction, participants described their participation in the eBook club program as a tool for managing negative affect and a source of emotional regulation. The act of reading, both individually and as part of a group, offered relief, stimulation, and a sense of psychological escape. One participant remarked, “When I read, I feel alive again… It’s kind of like a little escape for me” (Participant #3, 71 years). Another stated, “It was more about this comforting feeling… a kind of warmth and reassurance that lifted my mood” (Participant #14, 86 years). A participant also stated, “If I find I’m starting to think [negatively], I’ll pick up the Kobo and read… before I know it, I’m asleep” (Participant #9, 70 years).

#### 3.2.2. Theme 2: Selective Pruning of Social Networks

Another prominent theme corresponded to SST’s concept of selective pruning of social networks. Rather than seeking new or broad social networks, participants described forming emotionally rich and meaningful connections with their families and friends in the group. The small size of the reading circles facilitated an environment of intimacy, trust, and shared understanding. Participants consistently emphasized the depth of social connection rather than quantity. One participant explained, “We weren’t just talking about books; we were sharing our lives… It became more than a reading group, it was a place for connection and understanding” (Participant #9, 70 years). Another added, “Meeting with the ladies every week was something I really enjoyed… It was like we all showed up for each other” (Participant #11, 60 years). These experiences support SST’s assertion that, as individuals age, they increasingly invest in a smaller circle of emotionally rewarding relationships [55].

#### 3.2.3. Theme 3: Adaptive Coping

The final theme focused on how participants adapted to aging-related challenges while maintaining engagement in meaningful activities. SST posits that older adults strive to preserve emotionally fulfilling routines despite changes in physical or cognitive capacity. Participants in this study demonstrated this adaptive capacity by using technology, namely, Kobo eReaders, as a tool for maintaining independence and engagement. Participants described the eReaders as supportive tools that accommodated conditions such as arthritis, vision impairment, or memory challenges. As one participant noted, “I used to struggle with holding books… but the eReader changed everything. It brought back the simple joy of reading” (Participant #27, 81 years). Others highlighted how built-in features, such as automatic bookmarking, facilitated sustained reading: “Even with my memory issues, the Kobo helps me keep track… That gives me the confidence to keep reading” (Participant #14, 86 years). This adaptive engagement was closely linked to a desire for autonomy. The flexibility to read when and what they wanted allowed participants to exercise control over their routines. One participant shared, “I can pick up my Kobo anytime and read whatever I feel like… it gives me something meaningful I can hold onto” (Participant #5, 69 years). Another commented, “Being able to lose myself in a story even from my own home gives me a sense of independence and joy that I cherish” (Participant #10, 78 years). These reflections highlight how technological adaptation served as a mechanism for maintaining emotional engagement and motivation in later life.

## 4. Discussion

This pilot study evaluated the impacts of an eBook club, informed by SST, to mitigate apathy among community-dwelling older adults in three rural Canadian settings. The findings indicate a statistically significant reduction in apathy, supported by qualitative accounts that demonstrated increased emotional regulation, meaningful social interactions, and adaptive coping strategies. These findings align closely with the principles of SST [56] and extend the evidence based on shared reading programs for older adults. SST posits that as individuals age and perceive their remaining time as increasingly limited, their motivations shift from knowledge acquisition and novelty-seeking to emotionally meaningful, present-oriented goals. This theory provides a compelling framework for understanding why the eBook club contributed to a reduction in apathy among the participants.

Consistent with SST’s proposition that older adults preferentially seek emotionally gratifying experiences to optimize well-being [29,31], participants reported that reading emotionally resonant literature rekindled feelings of vitality, joy, and personal reflection. Participation in the eBook club offered immediate emotional satisfaction and simultaneously functioned as a tool for emotional regulation, helping to divert attention from negative thoughts and foster relaxation. These findings are consistent with prior research indicating that structured literary activities can enhance mood, promote emotional reflection, and contribute to psychological resilience in older populations [19]. The current findings extend this work by demonstrating that such benefits can also be achieved using accessible eReader technologies within a rural context.

In line with SST’s prediction that aging individuals prioritize emotionally rewarding social ties over expanding networks [57], participants emphasized the depth and authenticity of the relationships formed during the program. Weekly discussions provided opportunities for emotional sharing, trust-building, and mutual support, reinforcing previous findings from shared reading programs such as those documented by Milani et al. (2025) [58] and Kristensen et al. (2023) [27], both of which highlighted the social depth and therapeutic potential of group-based reading programs. These results suggest that group-based reading programs can serve as an effective platform for fostering selective, emotionally fulfilling social bonds among older adults, particularly in geographically isolated settings where social opportunities may otherwise be limited. It is noteworthy that while SST posits that older adults increasingly invest in a smaller circle of close, emotionally meaningful relationships, our findings indicate that participants were willing to form new connections within the context of a safe, structured group. Rather than contradicting SST, this may reflect a selective expansion of social networks to include new individuals who offer emotionally fulfilling interactions. The eBook club likely created an environment that felt emotionally secure and purpose-driven, encouraging even those with initially limited social engagement to selectively invest in new relationships.

The use of eReaders, for instance, allowed participants with arthritis, vision problems, or memory loss to continue reading and participating in discussions. Our findings are in tandem with Pihl et al. 2024 [59], where accessibility and adaptation were key to sustaining engagement among older adults. Moreover, the program’s emphasis on participant autonomy allowed individuals to maintain control over their engagement, reinforcing a sense of competence and emotional agency. These findings are consistent with the principles of SST, suggesting that older adults actively adapt their environments and strategies to sustain goal-directed emotional well-being even amidst functional decline [33]. They also align with broader evidence that technology, when accessible and meaningful, can serve as an important compensatory tool for supporting aging individuals’ psychological health [60].

All three participants with dementia in this study demonstrated either stable or improved apathy status following the eBook club program. Their qualitative feedback highlighted a capacity for meaningful participation when the activity was emotionally resonant, socially scaffolded, and cognitively accessible. These findings align with emerging research in dementia care demonstrating the efficacy of narrative- and emotion-centered programs. Studies by Billington et al., 2013 [19] and Longden et al., 2016 [61] showed that individuals with mild-to-moderate dementia derive substantial psychological benefit from structured literary engagement, particularly when the material is rich in emotional tone and delivered in a socially engaging context. Neuroimaging studies further support this by showing that default mode network activity (involved in autobiographical memory, identity, and narrative comprehension) remains relatively preserved in early dementia [62,63], potentially explaining why emotionally meaningful stories can still elicit engagement even in the presence of cognitive deficits.

Additionally, the findings of this study should be interpreted within the broader context of activity complexity versus emotional salience. Hughes et al. (2010) [64], in the MoVIES study, reported that reading newspapers, an activity likely low in emotional engagement and high in negatively valenced content, was associated with increased dementia risk, whereas emotionally meaningful, creative, or socially engaging activities such as crafts, hobbies, or reading fiction were protective against dementia. The eBook club, through its emphasis on shared emotional narratives and low-stress group dynamics, may thus have combined both affective relevance and neurocognitive accessibility, making it effective even for participants with cognitive impairment. This further reinforces SST’s claim that it is not the novelty or cognitive complexity of an activity that drives engagement in older age, but its alignment with emotionally grounded goals and the preservation of self-identity. The consistent improvement observed in both cognitively intact and cognitively impaired participants suggests that emotional engagement remains resilient in later life and can be meaningfully activated through well-designed cognitive-stimulating therapeutic programs [65].

Furthermore, technology plays a dual role as both an enabler and a potential barrier, making accessibility an important design element when implementing an eBook club among older adults. Previous research has shown that older adults often face challenges when adopting new technologies, including difficulties with interface design, learning new functions, and troubleshooting problems independently [66]. The unfamiliarity with digital devices can lead to frustration, suggesting the initial hurdles indicate that more robust support may be necessary during the onboarding process. However, in our study, many participants eventually overcame these challenges. Further, the challenges related to accessing free eBooks are consistent with previous studies, which noted that older adults often require simplified, step-by-step instructions to successfully engage with new technologies [67,68]. User-friendly eReaders or tablets with features such as high-contrast displays, large fonts, and audio narration can accommodate a wide range of needs. Providing hands-on technical support, including tutorials and troubleshooting, ensures participants feel confident using the devices [67].

Another noteworthy finding was the consistent preference among participants for fictional books. This preference aligns with predictions from SST that older adults increasingly prioritize emotionally meaningful experiences over knowledge acquisition or future-oriented goals [19]. Fiction, by nature, offers rich opportunities for emotional immersion, reflection, and vicarious social engagement, features that likely contributed to the emotional stimulation and regulation observed in this study. This is supported by Kristensen et al. (2023) [27], who found that narrative fiction fostered collective meaning-making and emotional expression in shared reading groups. The emphasis on fiction may also explain the observed shifts in apathy, as engagement with imaginative and emotionally resonant stories may have facilitated deeper psychological involvement and reduced motivational disengagement, resulting in a reduction in self-reported apathy.

### 4.1. Strengths and Limitations

To the best of our knowledge, this is the first study to explore the impact of the eBook club program on apathy among older adults in rural settings through the lens of SST. One of the key strengths of this study is its timing, as it was conducted in the immediate post-COVID-19 period. The study offered participants a valuable opportunity to reconnect socially and re-engage in meaningful activities, addressing both emotional and cognitive challenges that may have been exacerbated during the pandemic. Additionally, the use of eReaders and reading groups provided a flexible, accessible means for participants to interact, which was particularly beneficial considering the technological adaptations many had adopted during the pandemic. The timing of the study also allowed for insights into how digital tools can be used to foster social connections and reduce apathy among older adults in a post-pandemic world. While these findings are promising, several limitations must be acknowledged. Although the post-pandemic timing was a strength in terms of addressing social isolation during a period of limited engagement opportunities, it may also represent a contextual limitation. Thus, caution should be exercised when interpreting the intervention’s impacts. As a pilot study, the sample size was small, there was no control group, and the follow-up period was limited. The study also relied on self-reported measures of apathy, which may be subject to bias, and the brevity of this tool might have led to underestimation of the intervention’s impact. More comprehensive and objective measures, such as observational data, could provide further validation of the findings. Additionally, the sample was predominantly white, female, and community-dwelling, which may limit the generalizability of findings to other populations. Future research employing randomized controlled designs with larger, more diverse samples will help to further evaluate efficacy and generalizability. While SST emphasizes enduring emotionally meaningful relationships, our study primarily focused on short-term programs. Future longitudinal research should examine whether eBook clubs involving dyads/triads of older adults sustain benefits over time and whether they foster deeper emotional closeness across extended periods. Given that the group sessions were delivered in-person, older adults with greater mobility or geographic barriers may have been excluded, limiting reach to those most at risk of apathy. Findings related to participants with dementia are exploratory and should be interpreted with caution due to the small sample. Nevertheless, this study provides important preliminary evidence that SST-guided, emotionally meaningful programs can offer a viable strategy for addressing apathy and supporting emotional well-being among rural community-dwelling older adults.

### 4.2. Implications for Practice

This study demonstrates the potential impact of novel non-pharmacological approaches, such as eBook clubs, in reducing apathy among rural older adults. By aligning the program with the core tenets of socioemotional selectivity theory (SST), the findings provide preliminary evidence for the importance of fostering emotionally meaningful activities and close social connections to support psychological well-being and mitigate apathy in later life. As older adults increasingly prioritize present-focused and affectively rich experiences, programs that reflect these motivational shifts are more likely to promote sustained engagement and reduce emotional disengagement.

Practitioners working with older adults, especially those in rural or underserved settings, should consider incorporating accessible technologies such as eReaders to enable continued participation in activities that bring joy, even when physical or cognitive limitations are present. The use of lightweight, customizable digital reading devices in this study was well-received, offering an inclusive alternative to traditional books that may be difficult to manage for individuals with visual impairments, arthritis, or memory concerns. In addition, leveraging virtual platforms to connect residents with geographically distant close family may offer a feasible adaptation.

The emphasis on small group interactions in this program also reflects the importance of cultivating emotionally resonant relationships, rather than promoting broader but potentially superficial social networks. SST underscores that older adults benefit most from meaningful, supportive connections. eBook clubs designed with this principle in mind can become trusted spaces for emotional sharing, validation, and belonging.

To further enhance accessibility and inclusivity, peer support models [69] may be integrated into future program designs. In our study, participants frequently expressed appreciation for opportunities to learn from one another, particularly when navigating new technology. Encouraging more tech-savvy members to assist others not only reduces barriers to participation but also strengthens the sense of community and mutual support, elements essential to both emotional well-being and program sustainability. Future studies should also consider stratifying samples by cognitive diagnosis and using validated apathy instruments tailored for individuals with dementia. Moreover, incorporating neuropsychological assessments or functional neuroimaging may clarify whether observed reductions in apathy among cognitively impaired participants reflect genuine motivational re-engagement, improved affective responsiveness, or supportive effects of the social and environmental context.

## 5. Conclusions

This study provides preliminary evidence that participation in an eBook club is a feasible and adaptable non-pharmacological approach to mitigating apathy among older adults. By focusing on emotionally meaningful content and fostering selective, high-quality social relationships, the eBook club offered participants a sense of joy, purpose, and connectedness. The use of accessible digital technology further ensured that participants could maintain autonomy and emotional engagement despite physical or cognitive limitations. These findings highlight the importance of developing community-based programs that go beyond addressing functional needs to also support the deeper emotional goals that are prioritized in later life.

## Figures and Tables

**Table 1:** Sample characteristics (N = 28).

Characteristic	Category	Frequency (*n*)	Percentage (%)
Age	60–80 years	24	86%
	81+ years	4	14%
Gender	Woman	25	89%
	Man	3	11%
Education	High School	14	50%
	College/University	13	46%
	Other	1	4%
Marital Status	Married	16	57%
	Not Married	12	43%
Dementia Diagnosis	Yes	3	11%
	No	25	89%
Regular Reader	Yes	20	71%
	No	8	29%
Preferred Genre	Fiction	21	75%
	Non-Fiction/Other	7	25%
Use of Digital Reading Devices	Yes	9	33%
	No	19	67%
Prior Experience with eReaders	Yes	19	68%
	No	9	32%

**Table 2:** Pre- and post-program comparison of apathy scores (N = 28).

Measure	Pre-Program Apathy Scores	Post-Program Apathy Scores
Mean	1.54	0.64
Median	2.00	1.00
Standard Deviation	1.20	0.75
Interquartile Range (IQR)	2.00	1.00
*p*-value		< 0.001

Note: Scores range from 0 to 3 on the GDS-3A, with the highest scores indicating the highest levels of apathy.

## Data Availability

The data presented in this study are available upon request from the corresponding author. The data are not publicly available due to privacy and ethical restrictions.

## Supplementary Materials

The following supporting information can be downloaded at: https://www.mdpi.com/article/10.3390/geriatrics11010001/s1, Interview Guide.
