# Supplementary material for: Exploring the Impact of a Digital Reading Program on Apathy Among Community-Dwelling Older Adults in Rural Canada: Insights from Socioemotional Selectivity Theory
Source: Geriatrics (Basel). 2025 Dec 24;11(1):1. doi: 10.3390/geriatrics11010001 (PMC12821530; doi:10.3390/geriatrics11010001)
Supplement: Supplementary file 1 [file geriatrics-11-00001-s001.zip › geriatrics-3934682-supplementary.pdf]

Supplementary Material: Interview Guide

Demographic Information

1. How old are you? _________ years
2. What is your gender?

☐ Male
☐ Female
☐ Non-binary
☐ Prefer to self-identify, please specify: ___________________________________
☐ Prefer not to say

3. What is the highest education you have received?

☐ Less than high school, grade completed _______
☐ High School
☐ College or university degree
☐ Post-graduate studies, specify ________________

4. Have you been diagnosed with dementia?

☐ Yes
☐ No

5. What is your marital status?

☐ Never married
☐ Married
☐ Partner/significant other
☐ Widowed
☐ Separated
☐ Divorced

Post Interview

1. What motivated you to join the eBook club?
2. Can you share how the group experience impacted your mood, connection with others, or sense of purpose?
3. Did you feel a sense of connection with others in the group? If so, what contributed to that?
4. How often do you typically read, and is reading something you usually do alone or with others?
5. How did you experience using the eReader? Was it a barrier or a helpful tool for engaging with the group?
6. Did the shared reading and discussion feel emotionally meaningful or personally relevant to you? Why or why not?
7. Outside of this program, do you normally spend time with any of the group members? Or was this a new circle for you?
8. Was there anything about the group that helped you feel more or less emotionally connected or engaged?
9. Is there anything we haven't discussed that you would like to share about your experience in the program?
